# Niwaki Instead of Random Forests: Targeted Serial Sectioning Scanning Electron Microscopy With Reimaging Capabilities for Exploring Central Nervous System Cell Biology and Pathology

**DOI:** 10.3389/fnana.2021.732506

**Published:** 2021-10-13

**Authors:** Martina Schifferer, Nicolas Snaidero, Minou Djannatian, Martin Kerschensteiner, Thomas Misgeld

**Affiliations:** ^1^Center for Neurodegenerative Diseases (DZNE), Munich, Germany; ^2^Munich Cluster of Systems Neurology (SyNergy), Munich, Germany; ^3^Institute of Neuronal Cell Biology, Technical University of Munich, Munich, Germany; ^4^Hertie Institute for Clinical Brain Research, Tübingen, Germany; ^5^Institute of Clinical Neuroimmunology, University Hospital, Ludwig-Maximilians-University Munich, Munich, Germany; ^6^Faculty of Medicine, Biomedical Center (BMC), Ludwig-Maximilians-University Munich, Munich, Germany

**Keywords:** ATUM, array tomography, CLEM, volume EM, targeting, SEM

## Abstract

Ultrastructural analysis of discrete neurobiological structures by volume scanning electron microscopy (SEM) often constitutes a “needle-in-the-haystack” problem and therefore relies on sophisticated search strategies. The appropriate SEM approach for a given relocation task not only depends on the desired final image quality but also on the complexity and required accuracy of the screening process. Block-face SEM techniques like Focused Ion Beam or serial block-face SEM are “one-shot” imaging runs by nature and, thus, require precise relocation prior to acquisition. In contrast, “multi-shot” approaches conserve the sectioned tissue through the collection of serial sections onto solid support and allow reimaging. These tissue libraries generated by Array Tomography or Automated Tape Collecting Ultramicrotomy can be screened at low resolution to target high resolution SEM. This is particularly useful if a structure of interest is rare or has been predetermined by correlated light microscopy, which can assign molecular, dynamic and functional information to an ultrastructure. As such approaches require bridging mm to nm scales, they rely on tissue trimming at different stages of sample processing. Relocation is facilitated by endogenous or exogenous landmarks that are visible by several imaging modalities, combined with appropriate registration strategies that allow overlaying images of various sources. Here, we discuss the opportunities of using multi-shot serial sectioning SEM approaches, as well as suitable trimming and registration techniques, to slim down the high-resolution imaging volume to the actual structure of interest and hence facilitate ambitious targeted volume SEM projects.

## Introduction and Scope

Pioneering connectomics efforts have enabled advances in many steps of volume scanning electron microscopy (SEM), including sample preparation and image acquisition (Briggman and Bock, 2012; Kornfeld and Denk, 2018). These advances were driven by the need to upscale the amount of ultrastructural data that can be obtained. Developing commensurate analysis approaches to deal with the emerging large data volumes, such as “random forest” algorithms for segmentation, remain at the frontier of connectomics research (Berning et al., 2015; Januszewski et al., 2018; Scheffer, 2018; Schubert et al., 2019; Dorkenwald et al., 2020; Turner et al., 2020; Vergara et al., 2021). In comparison, tailored small-scale volume SEM of specific regions of interest resemble a “Niwaki” (Japanese for “sculpting trees”) task aimed at precision rather than high throughput. As equipment access and image analysis time are limiting in many EM laboratories, such small-scale volume SEM projects rely on trimming down the imaged tissue volume to target the actual structure of interest. Here we argue – illustrated by examples from neuroscience – that techniques developed in the context of connectomics if combined in the right order, can also greatly advance smaller scale EM projects in cell biology and pathology.

**FIGURE 1:**
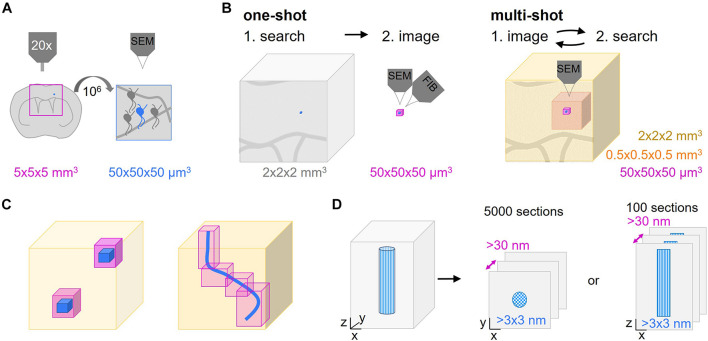
Multi-shot serial ultramicrotomy approaches for target relocation. **(A)** Schematic representation of the dimensions of a typical search task. The superficial parts of a brain region in a coronal central nervous system (CNS) slab can be visualized by light microscopy (pink frame, 5 × 5 × 5 mm^3^). The aim is to image the area of interest (blue neuron) which is a factor of 10^6^ smaller (50 × 50 × 50 μm^3^) by volume scanning electron microscopy (SEM). **(B,C)** Comparison of one-shot and multi-shot volume SEM methods. **(B)** A one-way search process that determines the region of interest (blue) as shown in panel **(A)** to be acquired by one-shot imaging like focused ion beam-SEM (FIB-SEM) (pink, max. 50 × 50 × 50 μm^3^). The remaining tissue is removed by trimming. In non-one-shot imaging the sections are screened by hierarchical imaging at different resolution levels (different shades of pink) from low to high. The lower the resolution the bigger the imaged volume that can be inspected. **(C)** Advantages of multi-shot imaging regarding the reduction of the actual high resolution imaging volume. The low resolution volume (light pink) is used to select the ROI(s) (blue) to be captured by high resolution imaging (magenta). **(D)** As automated tape-collecting ultramicrotomy (ATUM) does not provide isotropic high resolution voxels, the orientation of the structure of interest (blue patterned), either cross or longitudinally, has to be chosen carefully. This initial decision determines the high resolution imaging planes and the number of sections needed to cover the ROI.

The superior resolution of electron over light microscopy (LM) has proven indispensable not only for mapping connectivity, but also for detailed structural analysis of physiological and pathological processes that take place in the living nervous system. Only ultrastructural analysis can reveal many of the cellular, subcellular and membrane morphologies and at the same time provide an integration into the specific tissue environment – a density and breadth of information that even advanced LM approaches cannot deliver given the density of neuropil and the lack of intrinsic contrast. To generate the ultrastructural correlate of a developmental or disease-related process, specific structures within a particular central nervous system (CNS) volume have to be targeted. A typical experiment involves longitudinal *in vivo* imaging, such as 2-photon microscopy, of a genetically labeled CNS cell type. After fixation at a chosen endpoint, the sample is contrasted and embedded into resin – a process during which the ability to visualize the region of interest is lost and even careful positioning cannot avoid uncertainty about the relevant spatial coordinates. Hence, the search for a μm-scale structure within a CNS volume at the mm-scale resembles a “needle-in-the-haystack” problem, especially if a structure is has been singled out by precedent light microscopic observation or is rare *per se*. Typically, the structure of interest, e.g., a neuron, comprises 50 × 50 × 50 μm^3^, which has to be found within a tissue block that is 10^6^ times (5 × 5 × 5 mm^3^) larger (Figure 1A). Obviously, such a problem is simplified by reducing the “haystack”s’ size, i.e., by narrowing down the search volume based on larger and unique landmarks that define the immediate environment of the structure of interest. Then, in order to identify a preselected or rare event, this reduced volume has to be subjected to a suitable volume EM technique. For this, volume SEM has emerged as a powerful technique that can be used to efficiently analyze comparatively large tissue volumes if combined with a suitable sectioning approach. Volume SEM approaches can be classified into “single shot” techniques, where the tissue surface is imaged and then destroyed vs. “multi-shot” approaches, where serial sections of different thickness are collected onto solid support generating tissue libraries for repetitive and hierarchical imaging. The two most common “single shot” techniques are “serial block face” (SB-) SEM (Denk and Horstmann, 2004) and “focused ion beam” (FIB-) SEM (Knott et al., 2008), while the two predominant “multi-shot” techniques are ribbon-type sectioning called Array Tomography (AT) (Micheva and Smith, 2007) and automated tape-collecting ultramicrotomy (ATUM) (Schalek et al., 2011; Hayworth et al., 2014; Kasthuri et al., 2015). Notable, cross-over modalities that combine advantages of both approaches have also been suggested by others (Hayworth et al., 2015) and us (Kislinger et al., 2020).

Out of this smorgasbord of options, we here review approaches for targeted “multi-shot” volume SEM and provide recommendations for tapping their full potential. We describe options to approximate and search the targeted volume, discuss the use intrinsic and extrinsic landmarks, and delineate ideas on image registration. As disclaimers for the scope we chose for this review: First, volume EM is a fast developing field, therefore we apologize for any omission of emerging approaches. Second, when engaging in expensive and complicated analysis such as volume EM, all but the largest institutions will be constrained by available instrumentation and expertise, rather than following an idealized “best-of” workflow – a caveat that certainly constrains our perspective as well. Third, with sufficient effort and expertise, a given experimental aim can certainly be achieved by various technical means. So in sum, we are not arguing that the presented approach is the sole viable one in any situation. Still, we feel it is valuable to point out some advantages of the “multi-shot” approaches for studying neuronal and glial cell biology and “rare” cellular pathology, as these approaches originated in “connectomics”-style neuroscience (Helmstaedter et al., 2013; Kasthuri et al., 2015), as opposed to the complementary FIB-SEM approaches that have a stronger rooting in cell biology with recent developments toward high throughput (Xu et al., 2017; Hayworth et al., 2020). The latter have also received excellent coverage in recent reviews and original articles (Kizilyaprak et al., 2014; Narayan and Subramaniam, 2015; Karreman et al., 2016a; Luckner et al., 2018; Ronchi et al., 2021), to which we direct the interested reader.

## Classification of Volume Electron Microscopy Approaches

### Transmission Electron Microscopy Versus Scanning Electron Microscopy Techniques

Volume EM and connectomics pioneers in the last century have expanded classical sectioning for transmission EM (TEM) by collecting several hundreds or thousands sections on grids in a row (White et al., 1986). Although this serial sectioning TEM (ssTEM) technique has been successfully applied later on (Bumbarger et al., 2013; Bloss et al., 2018), it remains extremely tedious and requires great experience and special talent. Consequently, new ways of automation have been explored (Leighton, 1981) and refined (Denk and Horstmann, 2004). In the last 15 years, automation of volume EM for connectomics has been based on SEM instead of TEM (Kasthuri et al., 2015; Kornfeld and Denk, 2018), so SEM will be the focus of the following paragraphs. Still, also TEM has made major strides toward automation. Application of large-scale ssTEM was boosted by the development of a TEM Camera Array (TEMCA) for increased imaging throughput based on high-speed CCD or sCMOS cameras (Bock et al., 2011; Lee et al., 2016). Initially, single grids were loaded using a piezo-driven stage (Zheng et al., 2018), but recently a grid tape has been developed with slots for single ultrathin tissue sections (Yin et al., 2020). This collection method is very similar to the ATUM technique detailed below, but involves further automation for proper positioning of sections onto the slot position of the tape. While axial resolution is determined by section thickness in both “multi-shot” TEM and SEM, TEM reaches higher lateral resolution and signal-to-noise ratio (Zheng et al., 2018), while SEM tolerates thicker sections. In addition, for high-end ssTEM (Yin et al., 2020) and pixel-by-pixel multibeam SEM (Eberle and Zeidler, 2018) acquisition speeds are comparable (0.5–4 Gpixel/sec). So far, high-speed automated ssTEM equipment is only available in specialized facilities of the pioneering groups and is not the focus of this review (but see Zheng et al., 2018; Graham et al., 2019; Yin et al., 2020; Phelps et al., 2021).

### “One-Shot” Versus “Multi-Shot” Scanning Electron Microscopy Variants

“One-shot” volume SEM approaches include SB-SEM (Denk and Horstmann, 2004; Briggman et al., 2011; Helmstaedter et al., 2013; Mikula and Denk, 2015), FIB-SEM (Heymann et al., 2006; Knott et al., 2008; Sonomura et al., 2013), but also Gas Cluster Ion Beam SEM (GCIB-SEM) (Hayworth et al., 2020). Reimaging is impossible in these techniques as a section is irreversibly removed after imaging to approach the remaining block-face. Therefore, these methods require prior target localization (see section “Post-embedding Subdivision” μCT) with little correction options (Figure 1B). Even though a given region can be acquired only once, e.g., with FIB-SEM multiple similar regions within a block can be chosen for volume acquisition in order to comply with quantitative requirements (Rodriguez-Moreno et al., 2017; Serra Lleti et al., 2021). In contrast, the modular nature of “multi-shot” volume SEM entails the separation of physical sectioning from imaging. This enables repetitive acquisition rounds at different resolution (Wacker and Schroeder, 2013). The generated “tissue libraries” provide a screening platform for targeted volume SEM and also allow archiving tissue for subsequent analysis. Starting with the generation of a coarse map by low resolution prescreening at large horizontal field widths, even meandering or multiple regions of interest can easily be identified and selected for subsequent high resolution acquisition (Figure 1C; Wacker et al., 2018). This is especially relevant if the ultrastructural morphology of the target region or its exact location are not known *a priori* or not obvious, for example, in randomly distributed sites of local pathologies, such as the plaques in Alzheimer’s disease. Despite the impossibility for reimaging and archiving in FIB-SEM or SB-SEM, for a given region of interest similar targeting strategies can be used with these techniques (Heymann et al., 2006; Bosch et al., 2015; Blazquez-Llorca et al., 2017; Rodriguez-Moreno et al., 2017; Kikuchi et al., 2020; Ronchi et al., 2021). So while we are not explicitly referencing “single-shot” approaches below and rather focus on “multi-shot” techniques, many of the general points remain valid and beneficial for “one-shot” approaches, as described elsewhere (Karreman et al., 2016b; Lees et al., 2017b; Luckner et al., 2018; Kremer et al., 2021).

### Comparison of Different “Multi-Shot” Scanning Electron Microscopy Variants

**FIGURE 2:**
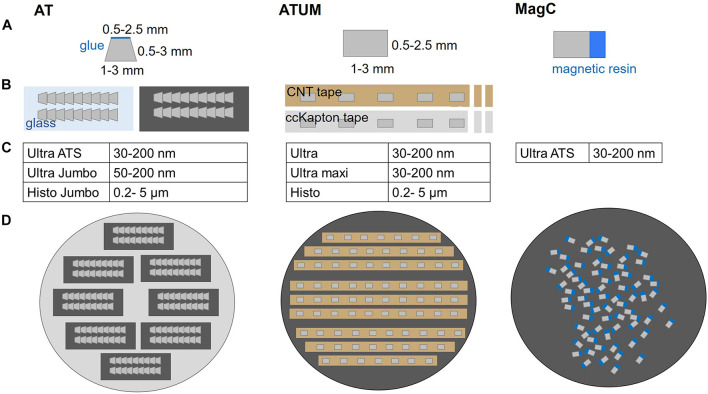
Comparison of multi-shot serial sectioning techniques. **(A)** Block face geometry and size, **(B)** schematics of sections on support, **(C)** diamond knife options and **(D)** mounted sections, ready for imaging are shown for each technique. Array Tomography (AT) generates ribbon-type sections that are assembled on glass or silicon. Typically, the block face shape is trapezoid with max. 2.5 mm edge length and the sections can be further adhered to each other by addition of glue to the upper edge. The ATUM approach features rectangular (or trapezoid) section collection onto carbon nanotube (CNT) or carbon coated Kapton (ccKapton) tape. Magnetic serial section collection (MagC) onto silicon wafers is based on random order attachment to a silicon wafer without additional support. The actual tissue resin blocks (gray) are attached to a magnetic resin block (blue).

Array Tomography or ATUM can equally be applied for a given targeting project and the choice mostly depends on the available equipment (Figure 2). In AT, 2–5 ribbons of 20–100 sections are collected and assembled on glass or silicon supports that roughly span 2 × 4 cm. The collection on transparent supports offers the advantage to perform post-embedding labeling followed by fluorescence microscopy. High pressure freezing and freeze substitution with low heavy metal concentrations are beneficial for preserving immunogenicity (Collman et al., 2015; Micheva and Phend, 2018) but for some epitopes chemical fixation with conventional embedding is sufficient (Micheva and Smith, 2007). The transfer of every ribbon and each support chip requires manual handling and thereby bears some risk of losing many sections at once (Wacker and Schroeder, 2013). On the other hand, ribbon sectioning ensures a reproducible orientation of successive sections and minimizes spatial separation. This is especially beneficial for mapping the sections in the image acquisition software as little focus adjustments are required during imaging (Kolotuev et al., 2010; Micheva and Phend, 2018; Wacker et al., 2020). In contrast, ATUM is based on the collection of series of single sections onto carbon nanotube (CNT; Kubota et al., 2018) or carbon-coated Kapton (Kasthuri et al., 2015) tape. Although occasional knife cleaning and water level adjustments are needed, the procedure is particularly stable and suited to collect thousands of sections (Hildebrand et al., 2017). However, the tape-collecting procedure requires subsequent tape assembly onto silicon wafers. Usually, the spacing between sections results in a lower density of sections per area compared to AT ribbons. This complicates the process of section mapping needed for serial imaging (Baena et al., 2019). Recently, magnetic serial section collection onto silicon wafers has been introduced with the aim to maximize the number of sections on a wafer (Templier, 2019; Figure 2). This comes with the drawback of losing the section order, thus introducing further mapping, acquisition and image analysis challenges.

### Challenges of Targeted “Multi-Shot” Volume Scanning Electron Microscopy

While we stress the potential of “multi-shot” volume SEM and ways to exploit it for different targeting tasks, it comes with some challenges and limitations. One major challenge of any targeted volume EM project is finding the structure of interest. The search strategy for the structure of interest depends on the tissue dimensions and the required specificity of the targeting approach, which together determine the probability to hit the region of interest. It is therefore important to decide for the most beneficial sectioning and imaging orientation already early on in a project. Lateral and axial relocation typically pose different challenges (Figure 1D). A large field of view and small sample depth are considered serial sectioning-friendly as this reduces the number of required sections. Specifically, trimming and sectioning determine the maximal z resolution, which is thereby fixed at an early stage. The exact lateral position can still be set at later stages thanks to hierarchical imaging and a large horizontal field width, usually spanning the whole tissue section (edge length typically 0.5–3 mm).

Another major drawback of “multi-shot” approaches is the irreversible determination of the axial resolution by the thickness of the section. Microtomy itself is intrinsically destructive and, consequently, the determination of the section thickness is a key decision at an early project stage. For example, a section thickness of 100 nm is sufficient to resolve groups of cells and thicker processes but it would not allow connectivity mapping in the CNS or the 3D visualization of organelles that could arise as a scientific question at a later stage of the project. So, if either isotropic high resolution voxels (<20 nm) are required or later limitations due to submaximal z resolution are to be avoided even at substantial cost of pre-hoc imaging time, FIB- or GCIB- SEM are the methods of choice.

In general, the cutting and collection process is the most vulnerable step in the AT and ATUM workflows. Folds are caused during sectioning or collection and have to be minimized by plasma discharge of the solid support material (Kasthuri et al., 2015) and support tissue surrounding the actual sample of interest (Hildebrand et al., 2017; Baena et al., 2019). In addition, microtomy is prone to variations in section thickness, rotation and stretching. Consequently, “multi-shot” techniques require more sophisticated image alignment techniques in comparison to “one-shot” block-face methods, a process that due to its imperfection can limit targeting precision. Indeed, even loss of sections can occur, which needs monitoring; if in a given project such loss could be catastrophic, considering a block-face alternative, where such intermittent losses are less likely, is worthwhile.

Further, while the possibility of re-imaging is the major advantage of “multi-shot” SEM, it also necessitates tissue contrasting with high heavy metal load in order to avoid beam damage. Suitable protocols include rOTO and (f)BROPA en bloc contrasting (Tapia et al., 2012; Mikula and Denk, 2015; Genoud et al., 2018). If the previously imaged area has been beam-damaged, its borders will become visible in subsequent images. Therefore, it is recommended to select the low-resolution field of views generously and image regions of interest within this area as a second step. Notably, TEMCA-based ssTEM methods largely overcome this beam-damage problem (Zheng et al., 2018; Yin et al., 2020).

## Complementary Imaging for Multi-Parametric Analysis and Correlation

Complementary imaging provides further information, particularly by increasing the field of view to create a map guiding a particular targeting approach.

### Pre-embedding Light Microscopy

Some targeting tasks in EM and obviously one-to-one correlation of a given structure in light and electron micrographs (correlated light and electron microscopy, CLEM), build on prior imaging with another imaging modality (Figure 1B). This typically involves either wide field, confocal or 2-photon microscopy (de Boer et al., 2015). Light microscopy can reveal characteristic landmarks that surround the region of interest (e.g., fluorescent cells or “negative” vasculature patterns based on tissue auto-fluorescence), as well as additional exogenous marks that further reduce the screening volume (Bishop et al., 2011; Begemann and Galic, 2016; Luckner et al., 2018). Usually, overview tile scans capture the tissue environment. This map can guide manual dissection using a binocular microscope in order to minimize the sample size of the actually embedded tissue (Snaidero et al., 2020).

### Post-embedding Imaging by μCT

Prior to sectioning, methods like X-ray micro-computed tomography (μCT) enable pre hoc navigation in embedded, osmium contrasted tissue blocks (Villani et al., 2019), thus revealing a low-resolution 3D view of the sample with the same contrast modality that will be used in EM. The intact sample is scanned at a voxel size down to one μm, revealing neuronal cell body distribution, myelination patterns or vasculature morphology. Recently, synchrotron-based X-ray tomography was applied in Drosophila specimen to achieve resolution of 15–20 nm for small and 0.5 μm for larger fields of view (Hwu et al., 2017; Fonseca et al., 2018). The strength of μCT-mediated targeting lies in its non-invasiveness. Also, the processed samples can be inspected to assess tissue shrinkage and distortion caused throughout the embedding procedure (Karreman et al., 2014). High resolution μCT thus allows the correlation with light microscopy data sets and the precise determination of the region of interest (Kuan et al., 2020). It is, however, limited to landmarks with high heavy metal staining in the μm range and provides coordinates in a virtual map rather than giving physical access to the region of interest. In practical terms, only few EM facilities can build on expensive and space-dominating μCT equipment, which currently prevents it from becoming a standard technique.

## Tissue Trimming

**FIGURE 3:**
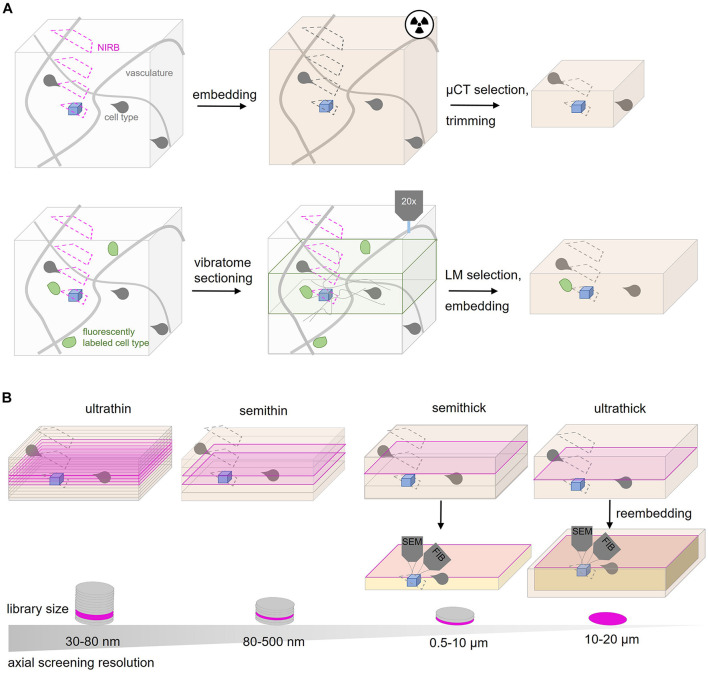
Tissue subdivision options for CLEM and rare events. **(A)** Screening strategies for CLEM or rare event targeting. The ROI (blue) is surrounded by a typical vascular pattern (gray lines) and specific cell types like glia (gray shapes). NIRBing (pink) at different axial positions can guide a correlation process if the same ROI from a LM experiment has to be relocated. μCT experiments (radiaton symbol) of the embedded tissue provides coordinates to guide ultramicrotomy-based trimming. Additional fiducials like genetically encoded proteins or dyes (green) can be used. Vibratome sectioning and LM-guided selection can reduce the volume for later EM inspection. **(B)** Ultramicrotomy options for screening for the ROI (blue). Ultrathin sectioning at 30–80 nm generates larger tissue libraries with many sections assembled on wafers compared to semithin (80–500 nm thickness), semithick (0.5–10 μm) or ultrathick (10–20 μm) sections. The tissue is inspected at the surface of each section (magenta) by SEM. Semithick and ultrathick sectioning enable coarse screening at the surface by SEM and subsequent targeted isotropic imaging of the ROI from the whole section thickness (orange). In contrast to semithick sectioning, ultrathick tissue partitioning requires reembedding.

Physical subdivision at the level of the fixed or embedded tissue reduces the actual screening volume and is therefore an important targeting step (Figure 3). Notably, this step is invariably “destructive,” no matter which imaging modality is later chosen, hence an efficient trimming strategy is key in any targeted volume EM project.

### Pre-embedding Subdivision

Tissue that is handed before fixation is vulnerable to artifacts due to cutting or stretching (Fix and Garman, 2000). Subdivision at this point implies an almost exclusive fixed sample approach, even if later high pressure freezing is desired, as sectioning fresh tissue tends to cause ultrastructural damage (Snaidero et al., 2017). Classical vibratome sectioning at a thickness of 50–200 μm exposes anatomical structures and fluorescent labels for light microscopic inspection (Li et al., 2011) to guide the relocation process and facilitates the penetration, e.g., of nuclear markers during post-fixation labeling. The imaging plane relative to the vibratome sectioning orientation can be preserved by a post-mortem holder that fixes the mouse head onto the vibratome stage (Luckner et al., 2018).

### Post-embedding Subdivision

Screening procedures can be further refined by subdivision of the embedded tissue. Historically, tissue trimming is combined with the generation of semithin sections (300–500 nm) that are stained and inspected by light microscopy (Pasquinelli et al., 1985; Dykstra and Reuss, 2003; Koga et al., 2015). In order to minimize the risk of removing relevant tissue regions, AT and ATUM provide the option to shift from such coarse trimming to collection of more and thinner sections (80–500 nm thickness). When approaching the target region, the section thickness can be reduced progressively to the desired axial resolution (30–200 nm). In general, the choice of section thickness is key to efficient screening and depends on the target region size, density in the tissue and desired resolution. The different thicknesses require diamond knifes that are suitable for the particular collection method (Figure 2). Ultrathin (30–80 nm; Kasthuri et al., 2015; Zonouzi et al., 2019), semithin (80–500 nm; Terasaki et al., 2020), semithick (0.5–10 μm; Kislinger et al., 2020) or ultrathick (20 μm; Hayworth et al., 2015) microtomy generate tissue libraries of decreasing section number at an increasingly coarse axial resolution (Figure 3).

Along this continuum, a recent variation is “semithick” sectioning, i.e., cutting at 0.5–10 μm (Baena et al., 2019; Kislinger et al., 2020). The advantage of this approach becomes apparent in the following example: in order to capture a complete larval zebrafish brain at 5.5 days post fertilization for EM analysis at 60 nm axial resolution, 17963 ultrathin sections had to be generated and collected on 68 m of tape assembled onto 80 wafers (Hildebrand et al., 2017). Semithick sectioning at 6 μm thickness would shrink the same zebrafish brain library to 200 sections fitting on a single wafer. Although this does not meet the requirements of connectivity mapping regarding the axial resolution, it would enable faster screening and isotropic imaging (see below) of a particular structure of interest (Figure 3).

### “Multi-Shot” Search Combined With “One-Shot” Imaging

Semithick and ultrathick sectioning generates small libraries and enables fast nm-scale screening by surface scanning. Notably, the remaining thickness below the accessible section surface can be further imaged by “one-shot” FIB-SEM microscopy (Hayworth et al., 2015; Kislinger et al., 2020). The axial resolution in FIB-SEM is not limited by microtomy as it uses a gallium ion beam for milling off the surface layer. Thus it generate volumes with high axial resolution below 30 nm (e.g., 3.7 × 3.7 × 20 nm; Merchan-Perez et al., 2009; Santuy et al., 2020) and even isotropic voxels where axial and xy resolution match (5 × 5 × 5 nm; Knott et al., 2008; Xu et al., 2017). Fast library screening and isotropic high resolution imaging are combined in this hybrid approach called ATUM-FIB (Kislinger et al., 2020). Serial semithick sections at a thickness between 2 and 10 μm are collected onto plastic tape and mounted onto a silicon wafer. The section surface is screened at SEM resolution in order to select particular regions. Selected sections can be remounted on SEM stubs for FIB-SEM milling which enables acquisition at isotropic voxels. ATUM-FIB is especially valuable for pre hoc searches in the sections themselves for rare objects of unknown ultrastructure or tissue distribution. A range of combinations of section thickness and number of FIB-SEM runs are possible to balance the project’s targeting needs, object dimension and fiducial density of with the fact that beam time at the FIB-SEM is often limited and the running costs for the gallium source typically are higher than tape or silicon support material.

## Landmarks

**FIGURE 4:**
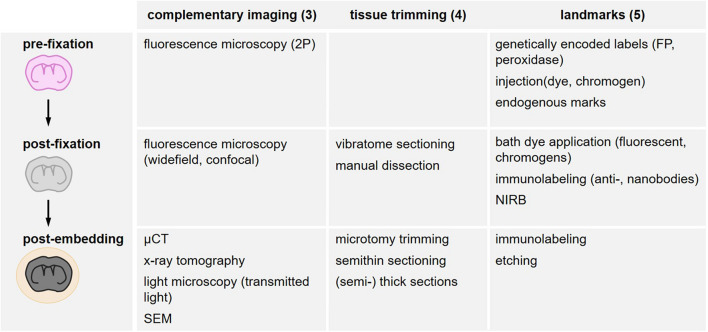
Overview of search strategies for the relocation of a region of interest. Different methods are structured according to their timing in the sample processing workflow as *in vivo* (pre-fixation), after fixative addition (post-fixation) and after resin embedding (post-embedding). Complementary imaging methods (“Complementary Imaging for Multi-Parametric Analysis and Correlation” section), tissue trimming strategies (“Tissue Trimming” section) and the variety of landmarks (“Landmarks” section) are listed accordingly.

Landmarks increase the probability to capture structures of interest within a tissue volume and preserve them for two- or three-dimensional acquisition. They need to be detectable in several imaging modalities and across scales (Figure 4) and permit to target trimming and screening, as well as finally relocating scarce subcellular objects within a tissue block of several mm^3^ (range: XY 1–3 mm and Z 0.3–2 mm). The different types of landmarks comprise endogenous landmarks (“Endogenous Landmarks”) and exogenously labeled biological structures further classified according to the method of labeling and when the label is introduced: genetically encoded labels (“Exogenous Tissue Branding”), pre-fixation labeling (“Pre-fixation Exogenous Labeling”), post-fixation labeling (“Genetically Encoded Labels”). These biological landmarks can be complemented by additional artificial exogenous marks (“Post-fixation Exogenous Labeling”), i.e., all types of non-biological fiducials (Figure 4).

### Endogenous Landmarks

Endogenous landmarks are non-labeled, tissue inherent biological structures that provide sufficient signals in complementary imaging modalities and SEM. In general, it is advisable to map endogenous landmarks by light microscopy methods in the living or fixed animal first, as such anatomical features can guide targeted trimming, especially in irregular tissue samples such as zebrafish larvae (Kolotuev et al., 2010; Durdu et al., 2014). Even the surface irregularities of vibratome sections or background autofluorescence from glutaraldehyde fixation can provide topological features that facilitate correlative workflows (Luckner et al., 2018). In CLEM approaches, autofluorescent structures like lipofuscin or extracellular fibers are valuable tissue-dependent landmarks. The vasculature or tracheae (in Drosophila), represent further informative sets of structures for coarse registration due to their electron lucent lumen in ultrastructural datasets, which can be exploited at low expense, e.g., by differential interference contrast (DIC) microscopy (Bock et al., 2011; Burgoyne et al., 2018; Luckner et al., 2018). Myelinated axons can be identified by spectral confocal reflectance microscopy (SCoRe), which exploits the characteristic reflected signals from multiple lasers of different wavelength at multilayered membranes (Schain et al., 2014).

### Exogenous Tissue Branding

Exogenous laser marks do not highlight a biological structure but are added to accentuate the tissue surrounding the region of interest – and could be considered the “in tissue” equivalent of artificial landmarks such as etched cover slips or bead decoration used for CLEM in cell culture (Kukulski et al., 2012; Hemelaar et al., 2017; Russell et al., 2017; Tanner et al., 2021). Near Infrared Branding (NIRB) marks can be burned into fixed tissue using high laser intensity line scans (most commonly using two-photon illumination). In confocal image stacks, these NIRB marks are visible as dark lines surrounded by auto-fluorescence, and in EM as sharply demarcated regions devoid of tissue (Bishop et al., 2011). NIRB marks are introduced around the imaged region at the particular z level (Maco et al., 2014; Karreman et al., 2016b; Snaidero et al., 2020). Asymmetric shapes and a series of marks at more superficial axial positions guide the trimming process toward the desired lateral position after fixation, during the ultramicrotomy and targeted imaging (Lees et al., 2017a). Additional laser etching after the embedding procedure preserves NIRB mark locations in the embedded tissue block for inspection in the SEM (Kolotuev et al., 2010).

### Pre-fixation Exogenous Labeling

Injection-mediated labeling of cells in living animals, e.g., using axonally transported dyes, has been introduced early during the advent of EM (Stoeckel et al., 1977; Bentivoglio et al., 1980). Since then, fluorescent or electron dense dyes have been widely exploited, especially in neurobiology (Vercelli et al., 2000). Horse radish peroxidase (HRP) was introduced for ultrastructural analysis, as it catalyzes the reaction of a chromogen [diaminobenzidine (DAB) or tetramethylbenzidine (TMB)] into an electron dense product (Straus, 1959; Kristensson and Olsson, 1971), thus allowing for light and electron microscopic examination. Applications include the study of blood brain barrier integrity (Brightman and Reese, 1969), anterograde tracing (Dietrichs and Walberg, 1979; Schönitzer and Holländer, 1981), as well as synaptic vesicle recycling (Heuser and Reese, 1973). Such approaches continue to remain valuable and often complementary approaches to the more recent genetic approaches (Papadopoulos and Dori, 1993).

### Genetically Encoded Labels

Genetically encoded fluorescent protein (FP) or electron density-generating tags are introduced into the living animal by viral approaches or by transgenic tagging. Genetic tagging provides homogenous molecular specificity independent of the tissue volume because the labeling is not limited by penetration depth. At the same time, selective or sparse electron dense labeling can facilitate image segmentation and volume reconstructions (Thomas et al., 2019). FP tags further allow longitudinal time-lapse imaging, thus providing dynamic information. Finally, genetic tags can serve as fiducial markers for correlation: FPs by direct image registration, if the underlying ultrastructural correlate exhibits specific shapes or electron dense patterns but in principle also by photo-oxidation of FPs to convert fluorescence into an electron dense signal (Grabenbauer, 2012). FP-induced peroxidation, however, tends to be inefficient and results in low signal-to-noise, so specific protein tags have been developed that efficiently generate electron dense signals. Photo-sensitizer efficiently generate reactive oxygen species for photo-oxidation (mini-SOG; Shu et al., 2011), while genetically encoded peroxidases enzymatically generate electron dense precipitates from chromogenic substrates (APEX, Martell et al., 2012; HRP, Li et al., 2010). Finally, metal-binding proteins [such as ferritin (Clarke and Royle, 2018)] have proven suitable to directly express an electron-dense label in specific cell types or subcellular structures in brain tissue (Joesch et al., 2016; Lin et al., 2016; Ng et al., 2016; Thomas et al., 2019). Combinations of these tags with specific genetic targeting techniques can provide additional information. For example, APEX labeling of different organelles can enable the multiplexed visualization of different cell types in one tissue (Zhang et al., 2019), while inducible expression allows pulse-chase experiments (Clarke and Royle, 2018). Drawbacks of peroxidase-based labeling include chromogen application, which is itself is penetration-limited and hence only applicable to smaller tissue volumes like vibratome sections. A further limit can be the delicate balance that needs to be achieved between general heavy metal contrasting and preservation of the specific label. Recently, discrete gold particle detection was combined with APEX labeling thereby improving signal detectability (Rae et al., 2021). However, similar to complementary attempts to genetically encode tags with characteristic electron dense geometries like iron-sequestering nano-compartments (Sigmund et al., 2019) or cysteine-mediated auto-nucleation of gold nanoparticles (Jiang et al., 2020), this approach is so far limited to cell culture applications.

### Post-fixation Exogenous Labeling

Cellular and subcellular structures can be labeled after fixation, usually on vibratome sections to improve tissue penetration. A simple form of post-fixation labeling is a nuclear marker staining (Hoechst, DRAQ5; Luckner et al., 2018). In order to target molecularly defined structures at the post-fixation stage, pre-embedding immune-labeling approaches are the method of choice. Classical immune-gold staining on vibratome sections provides discrete and specific signals with high precision, which is ideally suited for intracellular structures (Norris et al., 2017; Shibata et al., 2019; Sun et al., 2020). HRP coupled antibodies are typically preferred to render whole cells electron-dense as the spatial resolution of the signal is limited. The main advantage of HRP is that it requires very low quantity of antibody to achieve highly specific labeling of a particular cell type. While antibody penetration without permeabilization is restricted to the very surface (few μm) of vibratome sections, smaller nanobodies are detectable at 100 μm beyond the surface (Fang et al., 2018). Post-fixation labeling has to be carefully employed, as ultrastructural preservation may be compromised. Permeabilization constitutes the major hurdle for CLEM based on *en bloc* immunohistochemistry. Only recently, this problem has been circumvented by preservation of the extracellular space through increased extracellular osmolarity during chemical fixation (Pallotto et al., 2015; Fulton and Briggman, 2021).

## Imaging and Relocation Strategies

### Acquisition Sampling Options

Once coarse orientation has been achieved using endogenous and exogenous guides, targeting can be further refined at the level of image acquisition. Software for serial section mapping is available from Zeiss/Fibics (ATLAS) and Thermo Fisher Scientific (MAPS), as well as from non-commercial sources (Hayworth et al., 2014; Baena et al., 2019). The differences among these software packages, and general challenges related to their use have previously been discussed (Baena et al., 2019). With AT and ATUM, tile sets with large field of view can cover the entire section at a lateral resolution of 100–500 nm (Wacker et al., 2020). This enables the identification of coarse landmarks including somatic layers and vasculature. Screening for the right axial position can be achieved by acquiring every second to fifth section at low resolution. Even the sparse acquisition of only 2–5 sections per wafer (20–100x section thickness) can be beneficial if coarse tissue marks or guiding fiducials (such as large scale NIRB marks) are available to identify the larger sub-region bearing the structure of interest. Usually, another imaging round of a sub-region is acquired on every or every other section at medium resolution (10–100 nm) to finally pin-point the target. Only then, the actual high resolution (3–10 nm) stack is recorded.

## Registration Strategies

The relocation of an area of interest requires the registration of multimodal imaging data sets. As the scale-discrepancy between LM and EM data sets is huge, standard registration techniques building on image similarity are not applicable. If no μCT data is available, sample preparation and structural deformation that occurred between the acquisition of the LM and EM images constitute a further challenge. Consequently, non-rigid transformation is required, especially for chemically fixed tissue (Korogod et al., 2015). However, local warping can lead to registration errors. The alignment precision depends on the distribution, density and uniqueness of all extracted landmarks. Approaches to match graph structures independent of local appearance or global distance matrices have been developed (Fua and Knott, 2015). Currently, thin plate spline transformation is the method of choice, which uses landmark pairs to align LM and EM volumes (Hildebrand et al., 2017; Zheng et al., 2018). The regional target registration error can be optimized by adding more landmarks, especially in proximity to the structure of interest (Kukulski et al., 2011; Schorb and Briggs, 2014; Paul-Gilloteaux et al., 2017).

For connectomics analysis of functionally characterized brain regions, blood vessels and cell body patterns typically yield an alignment precision of about 5–10 μm (Drawitsch et al., 2018) for visual cortex and the retina (Bock et al., 2011; Briggman et al., 2011). In the mouse cortex, the coarse alignment by sparse labeling of nuclei and vasculature (30 μm distance) can be refined by characteristic electron-dense structures distributed at smaller average distances, e.g., myelinated axons (10 μm; Luckner et al., 2018). In cell culture CLEM experiments, artificial fiducials spaced at roughly 1–2 μm average distance reduces localization errors down to 50–100 nm (Kukulski et al., 2012). Spines or boutons (Cheng et al., 2019) are comparable high-density tissue landmarks (1 μm average distance) and can in principle be genetically tagged by FP fusions of pre- or postsynaptic structures. These examples illustrate that morphological uniqueness – in which CNS tissue is rich – is another important asset for high-precision registration.

Usually, both LM and EM 3D data sets and a potential μCT volume are co-registered (Luckner et al., 2018). Alternatively, or as a first approximation, maximum intensity projections of the *in vivo* fluorescence anatomy and evenly spaced electron micrographs can help render the 3D into a 2D correlation task (Bock et al., 2011). Additional (manual) skeleton tracings of smaller structures without characteristic contrast in the EM data set can facilitate registration (Briggman et al., 2011). The efficiency can be increased by a coarse segmentation of structures that do not resemble the ones on the LM template to create a “negative” background data set with structures different from the one of interest. Fast skeletonization of non-target structures helps to restrict the fine segmentation effort to the remaining candidates (Bates et al., 2019). Expanding this idea, FluoEM provides a two-step registration procedure by initial identification of the ultrastructural subvolume corresponding to the LM data set by coarse registration (Drawitsch et al., 2018). After all axons within an EM sub-volume of cortex have been completely skeletonized, the ones with the most similar geometry to the sparsely labeled LM features are computed. The required size of the sub-volume depends on the uniqueness or anisotropy of axon trajectories (40 μm length 90th percentile in mouse cerebral cortex). As a key decision factor, this compromises between computational costs and the need for further landmarks to yield a certain targeting precision (Drawitsch et al., 2018).

Software packages for registration of multimodal volume data sets include elastix (Klein et al., 2010; Shamonin et al., 2014), Amira (Karreman et al., 2014), 3D correlation toolbox (Arnold et al., 2016), BigWarp (Russell et al., 2017) or easy cell-correlative light to electron microscopy (eC-CLEM; Heiligenstein et al., 2017; Paul-Gilloteaux et al., 2017). The BigWarp (Hildebrand et al., 2017) and elmr (Zheng et al., 2018) software tools, which are now extended into the natverse platform (Bates et al., 2019), enable the integration of EM or CLEM data into public data repositories of other light level template drosophila or fish larval brains. Beyond the correlation among different imaging modalities, the information content of ultrastructural data can be enriched by registration onto spatial gene expression atlases (Vergara et al., 2021).

## Conclusion and Summary

Ultrastructural analysis of a discrete structure of interest does not necessarily require extensive high resolution volume SEM. Instead, large-scale acquisition can be circumvented by sophisticated targeting strategies that reduce the high resolution imaging volume to the area of interest. Thus, the ultrastructural volume does not have to be fully segmented (e.g., with “random forests” algorithms or similar “brute force” computational approaches), but can be tamed in a more hands-on “Niwaki” fashion. Multi-shot methods like AT and ATUM are especially suited for these targeting tasks as they preserve a tissue library that allows for hierarchical imaging at different resolution levels. Thereby, screening for the object of interest can be done at the nm resolution level. The section thickness at the initial microtomy step has to be chosen carefully, according to the desired axial resolution as well as the dimensions, distribution and frequency of a structure of interest. While one-shot volume SEM methods absolutely rely on coarse targeting methods at the mm (LM) and μm (μCT) scales, these strategies are also helpful in guiding multi-shot volume SEM searches. Complementary imaging of endogenous structures or exogenous markers saves acquisition, image analysis and correlation efforts. Obviously, availability of equipment and expertise have to be considered at any step. Building on these preconditions, a tailor-made multimodal approach with appropriate landmarks and physical or virtual sectioning methods can be designed. With increasing availability of public data sets and refinement of automated image analysis methods, registration bears a huge potential for the exploitation of ultrastructural information that is further elucidated by molecular identity and topology.

## Author Contributions

MS and TM contributed to conceptualization. MS wrote most of the manuscript and designed the figures with input from TM, NS, MD, and MK. All authors provided critical revision.

## Conflict of Interest

The authors declare that the research was conducted in the absence of any commercial or financial relationships that could be construed as a potential conflict of interest.

## Publisher’s Note

All claims expressed in this article are solely those of the authors and do not necessarily represent those of their affiliated organizations, or those of the publisher, the editors and the reviewers. Any product that may be evaluated in this article, or claim that may be made by its manufacturer, is not guaranteed or endorsed by the publisher.
